# Endocannabinoids and Inflammatory Response in Periodontal Ligament Cells

**DOI:** 10.1371/journal.pone.0107407

**Published:** 2014-09-16

**Authors:** Burcu Özdemir, Bin Shi, Hans Peter Bantleon, Andreas Moritz, Xiaohui Rausch-Fan, Oleh Andrukhov

**Affiliations:** 1 Department of Periodontology, Faculty of Dentistry, Gazi University, Ankara, Turkey; 2 Division of Oral Biology, Bernhard Gottlieb School of Dentistry, Medical University, Vienna, Austria; 3 Division of Orthodontics, Bernhard Gottlieb School of Dentistry, Medical University, Vienna, Austria; 4 Division of Conservative Dentistry, Periodontology and Prophylaxis, Bernhard Gottlieb School of Dentistry, Medical University, Vienna, Austria; 5 Department of Oral Surgery, First Affiliated Hospital of Fujian Medical University, Fuzhou, China; Faculty of Medicine & Health Sciences, United Arab Emirates

## Abstract

Endocannabinoids are associated with multiple regulatory functions in several tissues. The main endocannabinoids, anandamide (AEA) and 2-arachidonylglycerol (2-AG), have been detected in the gingival crevicular fluid of periodontitis patients, but the association between periodontal disease or human periodontal ligament cells (hPdLCs) and endocannabinoids still remain unclear. The aim of the present study was to examine the effects of AEA and 2-AG on the proliferation/viability and cytokine/chemokine production of hPdLCs in the presence/absence of *Porphyromonas gingivalis* lipopolysaccharide (*P. gingivalis* LPS). The proliferation/viability of hPdLCs was measured using 3,4,5-dimethylthiazol-2-yl-2,5-diphenyl tetrazolium bromide (MTT)-assay. Interleukin-6 (IL-6), interleukin-8 (IL-8), and monocyte chemotactic protein-1 (MCP-1) levels were examined at gene expression and protein level by real-time PCR and ELISA, respectively. AEA and 2-AG did not reveal any significant effects on proliferation/viability of hPdLCs in the absence of *P. gingivalis* LPS. However, hPdLCs viability was significantly increased by 10–20 µM AEA in the presence of *P. gingivalis* LPS (1 µg/ml). In the absence of *P. gingivalis* LPS, AEA and 2-AG did not exhibit any significant effect on the expression of IL-8 and MCP-1 expression in hPdLCs, whereas IL-6 expression was slightly enhanced by 10 µM 2-AG and not affected by AEA. In *P.gingivalis* LPS stimulated hPdLCs, 10 µM AEA down-regulated gene-expression and protein production of IL-6, IL-8, and MCP-1. In contrast, 10 µM 2-AG had an opposite effect and induced a significant up-regulation of gene and protein expression of IL-6 and IL-8 (P<0.05) as well as gene-expression of MCP-1 in *P. gingivalis* LPS stimulated hPdLCs. Our data suggest that AEA appears to have an anti-inflammatory and immune suppressive effect on hPdLCs’ host response to *P.gingivalis* LPS, whereas 2-AG appears to promote detrimental inflammatory processes. In conclusion, AEA and 2-AG might play an important role in the modulation of periodontal inflammation.

## Introduction

Endocannabinoid receptor ligands, also known as endocannabinoids are an emerging class of arachidonic acid derivates that activate cannabinoid receptors [1]. Expressions of major cannabinoid receptors, CB1 and CB2, have been documented in various immune cells and tissues [2], [3]. Anandamide (AEA) and 2-arachidonoylglycerol (2-AG) are major endocannabinoids, which behave as partial and full agonists at CB1 and CB2 receptors, respectively [1]. Endocannabinoids are known to be produced by various cell types, including endothelial cells, osteoblasts and osteoclasts [4]–[6].

The endocannabinoid (EC) system is strongly associated with an infection- or inflammation-related immune response [2], [3], [7]. Endocannabinoids are reported to modulate the proliferation and apoptosis of T- and B-lymphocytes, inflammatory cytokine production, and immune cell activation and migration [2], [8], [9]. The accumulating data on the EC system’s regulation of the immune response also identifies the EC system as a new therapeutic target for many diseases [2], [3], [7].

Periodontitis is a chronic inflammatory processes that occur in the periodontium in response to bacterial accumulations (dental plaque) on the teeth [10]. In periodontitis, tissue destruction is a result of an excessive inflammatory response, caused by the interaction of periodontal pathogenic bacteria with the host [11]. One of the major periodontal pathogens is *Porphyromonas gingivalis* (*P. gingivalis*), which belongs to the red complex group of bacteria [12]. Since the EC system is recognized to modulate immune response, an association between the EC system and periodontal inflammation is possible, but remains largely unknown.

To date, only limited number of studies have been examined the association between periodontal disease and the EC system [13]–[18]. Anandamide (AEA) and 2-arachidonoylglycerol (2-AG) were detected in the gingival crevicular fluid (GCF) of periodontitis patients [13], but not in periodontally healthy individuals [14]. Moreover, AEA levels appeared to decrease significantly in periodontitis patients after periodontal surgery, whereas 2-AG levels remained virtually unchanged [13]. *In vitro* studies show that AEA reduces the production of pro-inflammatory mediators induced by *P. gingivalis* lipopolysaccharide (LPS) in human gingival fibroblasts [14] and promotes the proliferation of these cells [13]. Animal studies have shown that AEA and its synthetic analog methanandamide diminish the inflammatory response in experimentally induced periodontitis [16], [17]. However, the exact role of endocannabinoid system in pathogenesis of periodontal disease remains largely unknown.

Human periodontal ligament cells (hPdLCs) are one of the essential elements for the homeostasis of the periodontal ligament, connecting the cementum to the alveolar bone. Along with various other cell types of periodontium, hPdLCs may be involved in the host response to periodontitis [19]. hPdLCs respond to *P. gingivalis* or its components by initiating an inflammatory response, including the expression and production of proinflammatory cytokines and chemokines, as shown both at the mRNA and protein levels [20]–[22]. hPdLC model stimulated with *P. gingivalis* LPS is widely used for simulating the *in* *vivo* conditions such as those found in diseased periodontal sites [18], [23]. The CB2 receptor is expressed in hPdLCs and its specific agonist is shown to attenuate inflammatory response to LPS [18]. However, it is not known how endocannabinoids AEA and 2-AG might influence the response of hPdLCs to periodontal pathogens.

In the present study, in order to determine the impact of AEA and 2-AG on the host response of human primary hPdLCs, the production of pro-inflammatory mediators, such as interleukin (IL)-6, IL-8, and MCP-1 in hPdLCs in response to stimulation with *P. gingivalis* LPS was examined.

## Materials and Methods

### Ethic Statement

Protocol for primary human periodontal ligament cells isolation was approved by the Ethics Committee of the Medical University of Vienna. Patients were informed in details before the surgical procedures and gave their written agreement.

### Cell Culture and Reagents

The hPdLCs were isolated from erupted third molars extracted from three healthy donors as described previously [24]. In addition, primary commercially available human periodontal ligament cells (Lonza, Switzerland) were used. None of the donors were smokers. hPdLCs were cultured in Dulbecco’s modified Eagle’s medium (DMEM, Gibco, Invitrogen, Wien, Austria) supplemented with fetal bovine serum (FBS, 10%), 50 µg/mL streptomycin, and 100 U/mL penicillin at 37°C under humidified atmosphere of 5% CO_2_ and 95% air. The hPdLCs from the third to fifth passages in culture were used.

Ultrapure LPS from *P. gingivalis* (Invivogen, California, USA) and single lots of AEA and 2-AG (both, Sigma-Aldrich Co, St. Louis, MO, USA) were used in the study. The AEA oil form was dissolved in 96% ethanol to a concentration of 10 mM. This stock solution was further diluted in 1% FCS containing DMEM to AEA concentrations of 0.1 to 20 µM. Therefore, the working solution with 20 µM AEA contained 0.192% ethanol. No effect of ethanol at this concentration on any parameters investigated was found. The 2-AG acetonitrile form stock solution was further diluted in 1% fetal calf serum (FCS) containing DMEM to 2-AG concentrations of 0.1 to 20 µM.

### Cell proliferation/viability

Cells were seeded in 48-well microplates (TPP, Trasadingen, Switzerland) at the density of 2×10^4^ cells in 300 µL of DMEM supplemented with 1% FCS with different amounts of AEA and 2-AG in the presence or absence of *P. gingivalis* LPS (1 µg/ml) and incubated for 24 h. After incubation 50 µl of 3,4,5-dimethylthiazol-2-yl-2,5-diphenyl tetrazolium bromide (MTT) reagent (5 mg/ml) (Sigma-Aldrich Co, St. Louis, MO, USA) were added to each well and cells were additionally incubated at 37°C for 2 hours. Subsequently, the media were discarded and 300 µL dimethylsulfoxide was added to each well, followed by 5 min incubation on a shaker. The absorbance was measured at 570 nm using a microplate reader (Molecular Devices, Sunnyvale, CA, USA).

### Measurements of cytokine production by hPdLCs

hPdLCs were seeded in 24-well plates at a density of 5×10^4^ cells/well in 500 µl of DMEM supplemented with 10% FCS. After 24 hours, the media were replaced by DMEM supplemented with 1% FBS and the hPdLCs were incubated by AEA/2-AG (1–10 µM) and/or *P. gingivalis* LPS (1 µg/ml). After 24 h incubation, the gene expression levels of IL-6, IL-8, and MCP-1 in the hPdLCs and the content of the corresponding protein in conditioned media were determined by qPCR and ELISA, respectively.

The mRNA expression levels of pro-inflammatory mediators were determined as described in previous studies [25], [26]. Isolation of mRNA and transcription into cDNA was performed using the TaqMan Gene Expression Cells-to-CT™ kit (Ambion/Applied Biosystems, Foster City, CA, USA) according to the manufacturer’s instructions. qPCR was performed on an ABI StepOnePlus device (Applied Biosystems, Foster City, CA, USA) in paired reactions using the TaqMan gene expression assays (Applied Biosystems, Foster City, CA, USA) with the following ID numbers: IL-6, Hs00985639_m1; IL-8, Hs00174103_m1; MCP-1, Hs00234140_m1; β-actin, Hs99999903_m1. qPCR reactions were performed in triplicate in 96-well plates using the following thermocycling conditions: 95°C for 10 min; 40 cycles, each for 15 s at 95°C and at 60°C for 1 min. The point at which the PCR product was first detected above a fixed threshold (cycle threshold, C_t_), was determined for each sample. Changes in the expression of target genes were calculated using the 2^−ΔΔCt^ method, where ΔΔC_t_ = (C_t_^target^−C_t_^β-actin^)_sample_−(C_t_^target^−C_t_^β-actin^)_control_, taking an untreated sample as a control.

Commercially available ELISA kits (Hoelzel Diagnostika, Cologne, Germany) were used for measurements of IL-6, IL-8, and MCP-1 levels in the conditioned medium. For measurement of IL-6 samples were not diluted, whereas for measurements of IL-8 and MCP-1 samples were diluted 1∶10 and 1∶5, respectively.

### Statistical analysis

After confirming normal distribution with the Kolmogorov-Smirnov test, the statistical differences between different groups were analysed by one-way analysis of variance (ANOVA) for repeated measures followed LSD post-hoc test. All statistical analysis was performed using the statistical program SPSS 19.0 (SPSS, Chicago, IL, USA). Data are expressed as mean ± s.e.m. Differences were considered to be statistically significant at p<0.05.

## Results

### Effect of Endocannabinoids on Proliferation/Viability of hPdLCs

**Figure 1:**
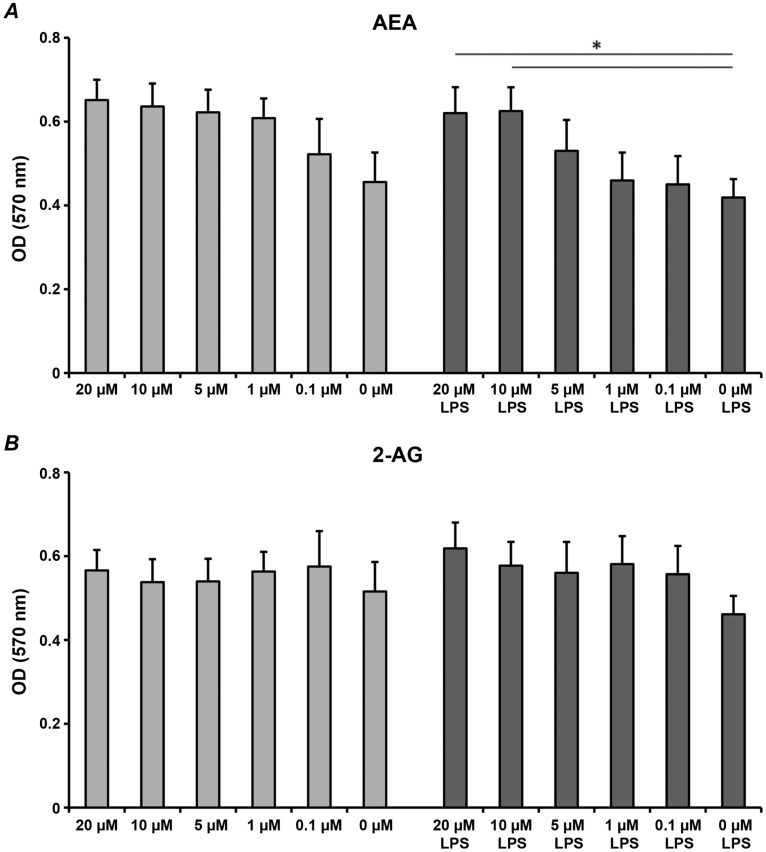
The effects of endocannabinoids on proliferation/viability of hPdLCs. hPdLCs were stimulated by different concentrations of AEA (A) or 2-AG (B) in the presence or in the absence of *P. gingivalis* LPS (1 µg/ml) for 24 h and the proliferation/viability was measured by the MTT method. Cells stimulated with DMEM supplemented by 1% FCS were used as a control (Co). The Y-axis represents mean ± s.e.m. of optical densities measured at 570 nm in 4 independent experiments. *Means were significantly different between groups (P<0.05).

The influence AEA and 2-AG in the concentrations of 0.1–20 µM on the proliferation/viability of hPdLCs after 24 hours of treatment is shown on the Figure 1. Neither AEA nor 2-AG exhibited significant effect on the proliferation/viability of hPDLC in the absence of *P. gingivalis* LPS. AEA in concentration of 10–20 µM induced a significant increase of hPdLCs proliferation/viability in the presence of *P. gingivalis* LPS (1 µg/ml). No effect of 2-AG on the proliferation/viability of hPDLCs in the presense of *P. gingivalis* LPS was observed.

### Effect of Endocannabinoids on Gene Expression of Pro-Inflammatory Mediators of HPdLCs

**Figure 2:**
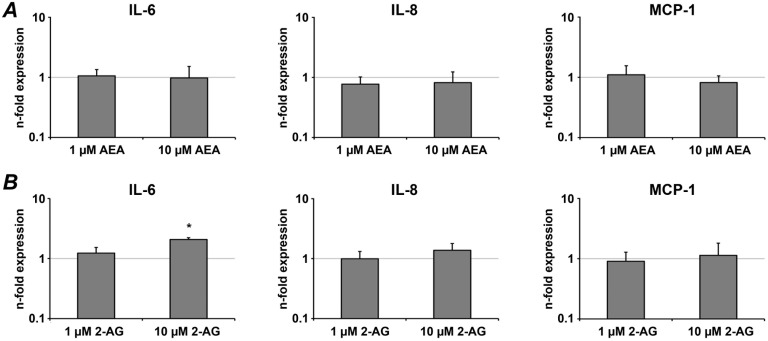
The effect of endocannabinoids on the expression of pro-inflammatory mediators in hPdLCs. hPdLCs were stimulated with AEA (A) or 2-AG (B) and the expression of pro-inflammatory mediators IL-6, IL-8, and MCP-1 was measured by real-time PCR. Changes in gene expression were calculated by 2^−ΔΔCt^ method, taking non-stimulated cells as a reference (2^−ΔΔCt^ = 1) and β-actin as a house-keeping gene. Each value represents the mean ± s.e.m of 4 independent experiments. *Means were significantly compared to the control group and tested with analysis of variance (P<0.05).

The effects of AEA and 2-AG on gene-expression levels of IL-6, IL-8, and MCP-1 in hPdLCs are summarized in Figure 2. Treatment of hPdLCs with AEA in concentration of 1–10 µM did not result in any significant effect on the gene expression levels of IL-6, IL-8, and MCP-1. Similarly, treatment of hPdLCs with 2-AG in concentration of 1–10 µM din not exhibit any significant effect on the gene expression levels of IL-8 and MCP-1. Treatment of hPdLCs with 10 µM 2-AG resulted in a significant increase of IL-6 gene expression level, whereas no significant changes were observed after treatment with 1 µM 2-AG.

### Effect of AEA on pro-inflammatory mediators levels in hPdLCs stimulated by *P. gingivalis* LPS

**Figure 3:**
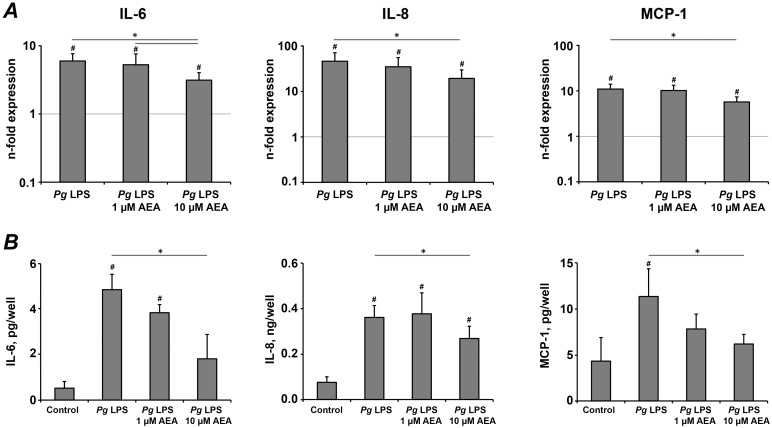
The effect of AEA on the production of pro-inflammatory mediators in hPdLCs in response to stimulation with *P. gingivalis* LPS. hPdLC were stimulated with *P. gingivalis* LPS in the presence or in the absence of AEA for 24 h, and the production of pro-inflammatory mediators was measured on gene and protein levels by real-time PCR (A) and ELISA (B) respectively. A – Changes in the gene expression levels of IL-6, IL-8, and MCP-1 calculated by 2^−ΔΔCt^ method taking non-stimulated cells as a reference (2^−ΔΔCt^ = 1) and β-actin as a house-keeping gene. B – Content of pro-inflammatory mediators in the conditioned media measured by ELISA. Each value represents the mean ± s.e.m of 4 independent experiments. ^#^Means were significantly compared to the control group, and tested with analysis of variance (P<0.05). *Means were significantly different between groups, tested with analysis of variance (P<0.05).

In next series of experiments, we investigated the effect of AEA on the hPdLCs response to *P. gingivalis* LPS. *P. gingivalis* LPS induced a significant increase of the expression of IL-6, IL-8, and MCP-1 on both gene and protein levels (p<0.05). 10 µM AEA induced a significant decrease of *P. gingivalis* LPS stimulated IL-6, IL-8 and MCP-1 gene expression levels (Fig. 3A, p<0.05). In agreement with qPCR data, 10 µM AEA induced a significant decrease in the content of IL-6, IL-8, and MCP-1 in the conditioned media (Fig. 3B, p<0.05). AEA in concentration 1 µM had no significant effect on the response of hPdLCs to *P. gingivalis* LPS.

### Effect of 2-AG on pro-inflammatory mediators levels in hPdLCs stimulated by *P. gingivalis* LPS

**Figure 4:**
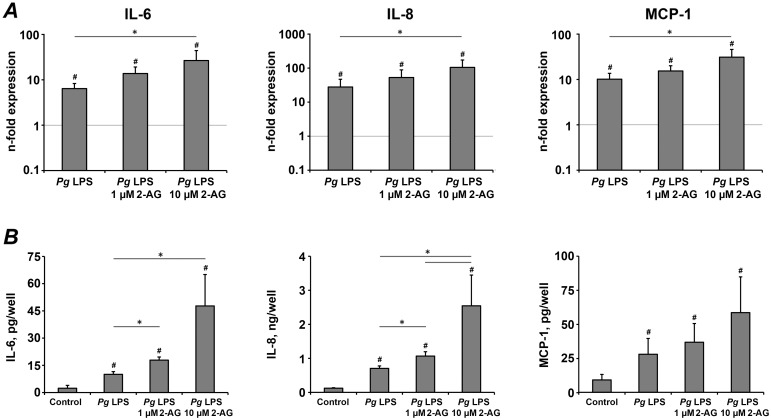
The effect of 2-AG on the production of pro-inflammatory mediators in hPdLCs in response to stimulation with *P. gingivalis* LPS. hPdLC were stimulated with *P. gingivalis* LPS in the presence or in the absence of 2-AG for 24 h and the production of pro-inflammatory mediators was measured on gene and protein levels by real-time PCR (A) and ELISA (B) respectively. A – Changes in the gene expression levels of IL-6, IL-8, and MCP-1 calculated by 2^−ΔΔCt^ method taking non-stimulated cells as a reference (2^−ΔΔCt^ = 1) and β-actin as a house-keeping gene. B – Content of pro-inflammatory mediators in the conditioned media measured by ELISA. Each value represents the mean ± s.e.m of 4 independent experiments. ^#^Means were significantly compared to control group, tested with analysis of variance (P<0.05). *Means were significantly different between groups, tested with analysis of variance (P<0.05).

In last series of experiments, we investigated the effect of 2-AG on *P. gingivalis* LPS induced response in hPdLCs. 10 µM 2-AG induced a significant increase of *P. gingivalis* LPS stimulated IL-6, IL-8 and MCP-1 gene expression levels (Fig. 4A, p<0.05), whereas no significant effect of 1 µM 2-AG was observed. Measurements of proteins content in the conditioned media showed that the *P. gingivalis* LPS stimulated IL-6 and IL-8 production by hPdLCs were significantly increased by 1–10 µM 2-AG in a concentration-dependent manner (Fig. 4B, p<0.05). In contrast, no significant effect of 2-AG on *P.gingivalis* LPS induced MCP-1 production by hPdLCs was observed (Fig. 4B, p>0.09).

## Discussion

To our knowledge, this study is the first to evaluate the effects of the endocannabinoids AEA and 2-AG on the production of pro-inflammatory by hPdLCs. In particular, we investigated the effect of endocannabinoids on proliferation/viability as well as on the production of IL-6, IL-8, and MCP-1 by hPdLCs. IL-6 is a multifunctional pro-inflammatory cytokine that plays a significant role in inflammation and also stimulates osteoclastogenesis and bone destruction in chronic inflammatory diseases such as periodontitis [27]. One of the most important chemotactic factors for polymorphonuclear leukocytes (PMNLs) is IL-8 [28], which is also produced by hPdLCs, and its levels have been shown to increase in the presence of *P. gingivalis* LPS [29]. MCP-1 chemokine is a chemo-attractant for macrophages and their precursors, monocytes, and a small subset of lymphocytes [30]. Earlier, GCF MCP-1 levels were associated with periodontitis [31].

AEA and 2-AG are reported to have different effects on the proliferation/viability of different cell types [13], [14], [32]. In the present study we used MTT assay, which detects the activity of the mitochondria respiratory chain and is considered as a measure for proliferation of viable cells [33]. Our current data suggests that AEA or 2-AG in concentrations of up to 20 µM did not have significant effects on the proliferation/viability of hPdLCs in the absence of *P. gingivalis* LPS (Fig. 1). However, in the presence of *P. gingivalis* LPS AEA induced a significant increase in hPdLCs proliferation viability. This increase could be associated with activation of CB1 and CB2 receptors. Previous study on human gingival fibroblasts show that AEA as well as specific agomists of CB1 and CB2 receptors induce an increase in cell proliferation [13]. Another study shows that *P. gingivalis* LPS at high concentrations (10 µg/ml) stimulates proliferation of hPdLCs [34]. In our study we used *P. gingivalis* LPS in lower concentration (1 µg/ml) but it can be assumed that the increase in proliferation/viability might be due to cumulative effect of LPS and AEA.

Our results revealed that AEA has suppressive effects on the pro-inflammatory mediators production triggered by *P. gingivalis* LPS. 10 µM AEA significantly reduced the gene-expression levels of all investigated pro-inflammatory mediators, as well as their levels in conditioned media. A similar effect of AEA on the inflammatory response is also found in other cell types. Particularly, the production of IL-6, IL-8, and MCP-1 is shown to be significantly reduced by 10 µM AEA in *P. gingivalis* LPS stimulated human gingival fibroblasts [14]. The production of IL-6 by murine macrophages in response to *Escherichia coli* LPS stimulation is shown to be significantly reduced by 10–30 µM AEA [35]. The effect of AEA on the production of pro-inflammatory mediators is concentration-dependent: 1 µM AEA did not exhibit any significant effect on the response of hPdLCs to *P. gingivalis* LPS stimulation. This finding is in agreement with a previous study reporting no significant effects of 1 µM AEA on MCP-1 production of HL-60 cells with or without stimulation by *E. coli* LPS [36].

2-AG stimulation induced an increase of the *P. gingivalis* LPS stimulated production of pro-inflammatory mediator by hPdLCs. 10 µM 2-AG caused a significant increase of IL-6 and IL-8 gene expression levels and protein release by hPdLCs. Moreover, the content of IL-6 and IL-8 in hPdLCs conditioned media were also increased also by 1 µM 2-AG. The gene-expression levels of MCP-1 after stimulation with *P. gingivalis* were significantly increased in the presence of 10 µM 2-AG but no significant differences was observed on protein level. The differences between qPCR and ELISA experiments observed in our study could be due to the fact that qPCR measurements reflect gene expression levels exactly after 24 h treatment, whereas amount of protein in conditioned media reflect accumulation during whole treatment period. Studies on other cells controversially describe the effect of 2-AG on inflammatory response. Stimulation of human promyelocytic leukemia cells with 1 µM 2-AG alone or together with 100 ng/ml of *E. coli* LPS were reported to increase IL-8 and MCP-1 levels significantly [36]. Contrariwise, 3–30 µM 2-AG was reported to inhibit LPS induced IL-6 production in murine J774 macrophages in a concentration-dependent manner [35].

Present data revealed that AEA has no substantial influence on the expression of pro-inflammatory mediators in the absence of *P. gingivalis* LPS stimulation. We also did not find any significant effect of 2-AG on the expression of IL-8 and MCP-1 in hPdLCs without *P. gingivalis* LPS stimulation. In contrast, 2-AG itself induced a significant increase of IL-6 gene expression in hPdLCs. However, this effect was rather small compared to that of *P. gingivalis* LPS response itself and to the 2-AG induced augmentation of the *P. gingivalis* response. These observations suggest that endocannabinoids are probably not able to induce pro-inflammatory response themselves, but rather affect some pro-inflammatory signalling pathways in hPdLCs and modulate inflammatory response of these cells.

Cytokine production by hPdLCs upon LPS stimulation is thought to play an important role in the progression of periodontal disease. In our experiments, stimulation of hPdLCs with *P. gingivalis* LPS resulted in the increase of IL-6, IL-8, and MCP-1 expression. However, some quantitative differences in the production of pro-inflammatory mediators upon *P. gingivalis* LPS treatment were observed (see, Figure 3 and 4). There are several reasons, which can underlie these differences. First, hPdLC in culture are supposed to consist of several subpopulations [19]. Second, large inter-individual heterogeneity in hPdLCs response to *P. gingivalis* exists [21]. Third, properties of hPdLCs might be changed with passaging [37]. Nevertheless, it is unlikely that these quantitative differences in hPdLCs response to *P. gingivalis* LPS might influence the conclusion of our study on the effect of AEA and 2-AG on IL-6, IL-8, and MCP-1.

So far not much clinical data has been published about endocannabinoids in periodontology. AEA and 2-AG were detected in the GCF of periodontitis patients [13], [14]. Kozono et al. examined the role of the EC system in periodontal wound healing by GCF analysis of patients with periodontitis before and after periodontal surgery, and demonstrated that AEA levels significantly increased 3 days after periodontal surgery in GCF, whereas 2-AG levels did not change significantly [13]. Our present results suggest that both endocannabinoids AEA and 2-AG have significant effects on the regulation of host response from hPdLCs triggered by *P. gingivalis* LPS. While neither endocannabinoid had an important effect on cell proliferation/viability of hPdLCs, it was interesting to see that they seemed to have opposite effects on IL-6, IL-8 and MCP-1 levels of *P. gingivalis* LPS induced hPdLCs. Immune response in periodontitis is aimed to destroy and eliminate periodontal pathogen, but excessive response leads to host tissue destruction [38]. Regulation of cytokine production by endocannabinoids might play an important role in the balancing between bacterial clearance and tissue destruction in periodontal disease. Our data reveals that AEA may have an anti-inflammatory and immune suppressive effect on host defense of *P. gingivalis* LPS induced hPdLCs, whereas 2-AG seems to support inflammatory processes. Regulatory mechanisms beyond these opposite effects on cytokine and chemokine production still remain unknown. Moreover, it has also been stated that stressful or pathological inducements affect the EC system within a certain tissue in more than just one way, and end up with more than just one functional result depending on the nature and duration of this inducement [39].

The reasons why AEA and 2-AG exert opposite effects on LPS-induced production of pro-inflammatory cytokines in hPdLCs are not entirely clear. Both AEA and 2-AG are described as agonists for both CB1 and CB2 receptors [1], [40]. However, there are conflicting results about their affinity to these receptors [1]. In addition, AEA but not 2-AG is known to activate transient receptor potential vanilloid type-1 receptor (TRPV1) [41]. Recent study shows that this receptor is expressed in hPdLC and might be activated by AEA [42]. Moreover, ablation of TRPV1 in mice results in exacerbated inflammatory response during endotoxic shock [43], which also suggests the role of this receptor in LPS-induced signaling. Therefore, it could be hypothesized that activation of TRPV1 by AEA can underline reduced inflammatory response of hPdLC upon stimulation with *P. gingivalis* LPS.

The endocannabinoid system is one of the most important signalling systems that has been discovered within the last few decades, and many researchers are currently focused on it for developing new treatment strategies for many disease and pathological conditions [39]. We still assume that, in the near future, regulation of the endocannabinoid system might play a role in the management of periodontal disease. Further studies, clinical as well as experimental, are needed to understand the balance between AEA and 2-AG and their exact roles in periodontal inflammation and regeneration.
